# Effects of Rhythm Control for Atrial Fibrillation on Cardiac Remodeling and Valvular Regurgitation in Patients with Heart Failure

**DOI:** 10.1007/s10557-023-07489-2

**Published:** 2023-08-30

**Authors:** Jinping Si, Zijie Ding, Xuefu Chen, Lin Bai, Yuxi Sun, Xinxin Zhang, Yanli Zhang, Yunlong Xia, Ying Liu

**Affiliations:** 1 Department of Cardiology, The First Affiliated Hospital of Dalian Medical University, 193 United Road, Dalian, 116021 Liaoning Province China; 2 Department of Cardiology, West China Hospital, Sichuan University, Chengdu, 610041 Sichuan Province China

**Keywords:** Rhythm control, Atrial fibrillation, Heart failure, Cardiac remodeling, Functional regurgitation

## Abstract

**Purpose:**

Previous studies investigating cardiac remodeling and functional regurgitation of rhythm control for atrial fibrillation (AF) in heart failure (HF) are limited. Therefore, this study aimed to evaluate the impact of rhythm control for AF on cardiac remodeling and functional regurgitation in the spectrum of HF. Its effect on prognosis was explored.

**Methods:**

According to the treatment strategies of AF, the cohort was classified into the rhythm control and rate control groups. To further detect the implications of rhythm control on cardiac remodeling, functional regurgitation, and outcomes in HF subtypes, patients were further divided into HF with reduced ejection fraction (HFrEF), HF with mildly reduced ejection fraction, and HF with preserved ejection fraction (HFpEF) subgroups.

**Results:**

A total of 828 patients were enrolled, with 307 patients in the rhythm control group and 521 patients in the rate control group. Over a median follow-up time of 3.8 years, patients with rhythm control treatments experienced improvements in biatrial structure parameters, left ventricular ejection fraction, and functional regurgitation (mitral and tricuspid regurgitation) compared with rate control treatment (*p* < 0.05). Cox regression analysis demonstrated that rhythm control reduced the risks of all-cause mortality (HR 0.436 [95% CI, 0.218–0.871], *p* = 0.019) in HFpEF and HF-related admissions in HFrEF (HR 0.500 [95% CI, 0.330–0.757], *p* = 0.001) and HFpEF (HR 0.541 [95% CI, 0.407–0.720], *p* < 0.001); these associations were similar after adjusting for multiple confounders.

**Conclusions:**

Rhythm control therapy can be considered an appropriate treatment strategy for the management of AF in HF to improve cardiac remodeling, functional regurgitation, and prognosis.

## Introduction

Heart failure (HF) is a heterogeneous syndrome; its main pathophysiological mechanism is left ventricular (LV) remodeling, which manifests as changes in LV quantity, size, structure, and composition of the extracellular matrix [1]. HF is currently divided into three categories based on baseline left ventricular ejection fraction (LVEF): HF with reduced ejection fraction (HFrEF) (LVEF ≤ 40%), HF with mildly reduced ejection fraction (HFmrEF) (LVEF 41–49%), and HF with preserved ejection fraction (HFpEF) (LVEF ≥ 50%) [2]. HF and atrial fibrillation (AF) often co-exist through shared common risk factors [3, 4]. They are also bidirectionally related, where AF can precipitate HF but can develop as a consequence of HF and cardiomyopathy [5]. Importantly, the development of AF could induce biatrial remodeling and enlargement [6]. In addition, AF may also lead to ventricular remodeling and HF by several mechanisms [7]. As is well-documented, the coexistence of AF with HF is associated with a worse prognosis [8].

Medical and device therapies may prevent this pathophysiologic process and perhaps reverse cardiac remodeling. Indeed, existing clinical trials have reported the benefits of AF rhythm control on myocardial function and prognosis in patients with HF [9–11]. Consequently, the 2022 HF guidelines provide a class II recommendation for catheter ablation (CA) in patients with AF and HF [2]. However, the role of rhythm control in improving cardiac remodeling, including left atrium (LA), LV, right atrium (RA), and right ventricle (RV) across the spectrum of LVEF functions, remains elusive. Moreover, atrial enlargement from AF also results in annular enlargement and atrial functional mitral regurgitation (FMR)/functional tricuspid regurgitation (FTR), which further exacerbates cardiac function [12]. Nevertheless, there are limited data on the effect of rhythm control for AF on FMR and FTR in patients with HF. The purpose of this study was to evaluate the efficacy of rhythm control on cardiac structure remodeling, functional atrioventricular regurgitation, and prognosis across the spectrum of HF.

## Methods

### Study Population

This was a retrospective, single-center, observational, real-world study. Patients diagnosed with HF and AF who were hospitalized at The First Affiliated Hospital of Dalian Medical University between January 1, 2012, and December 30, 2020, were involved in this study. Patients were excluded if they: (1) were aged > 80 years; (2) were missing echocardiogram or electrocardiogram (ECG) results; (3) had organic valve disease; (4) had previously undergone valvular surgery; (5) were lost to follow-up; (6) had an estimated life expectancy of less than 1 year; (7) suffered from end-stage renal failure.

### Treatment Strategies for AF

The rhythm control therapy for AF comprised CA and electrical cardioversion (ECV) (initial energy 100 J or 150 J). All the patients received a direct oral anticoagulant drug or warfarin (target international normalized ratio of 2–3) for at least 3 weeks before and 2 months after the procedure. Intracardiac thrombus was excluded via transoesophageal echocardiography prior to the procedure. Preoperative and postoperative administration of amiodarone was required for patients (postoperative prescription: 600 mg/d for the first week, 400 mg/d for the second week, 200 mg/d for the third week, and treatment thereafter was at the discretion of the treating physician). LA CT was performed to guide transseptal puncture and anatomical orientation. Intravenous heparin was administered to maintain an activated clotting time of 250–350 s during the CA procedure. A circular pulmonary vein (PV) mapping catheter (LASSO, Biosense Webster, Irvine, USA) and a saline-irrigated ablation catheter (Termocool SMART TOUCH SF, Biosense Webster, Irvine, USA) were used for mapping and ablation, respectively. Radiofrequency energy was delivered with a limit of 35 W (infusion rate 22 mL/min) at the target catheter tip at a temperature of 45 ºC. For paroxysmal AF, the goal of the ablation procedure was to achieve complete PV isolation, manifested as the absence of PV potentials or PV-left atrial conduction. As for persistent AF, the main CA procedure was PV isolation; additional ablation procedures were performed at the operator’s discretion, including ablation of complex fractionated atrial electrograms, creation of linear lesions, or combinations thereof.

Patients received rate control therapy (beta-blockers/digoxin/diltiazem) to achieve a resting ventricular rate of < 80 beats/min and < 110 beats/min during moderate exercise.

Notably, all enrolled patients received guideline-directed medical therapy during hospitalization.

### Clinical Data

Baseline demographics, laboratory data, echocardiogram data, medical history, and medications were recorded. Echocardiogram data predominantly focused on the following parameters: (1) RA superior–inferior diameter (RASID); (2) RA transverse diameter (RATD); (3) LA superior–inferior diameter (LASID); (4) LA transverse diameter (LATD); (4) LA volume (LAV); (5) LAV index (LAVI); (6) LV end-diastolic dimension (LVEDD); (7) RV end-diastolic dimension (RVEDD); (8) LVEF; (9) FMR/FTR severity. FMR/FTR severity was graded as none, mild, mild-moderate, moderate, moderate-severe, or severe by imaging cardiologists according to the American Society of Echocardiography guidelines [13]. In this cohort, all patients underwent at least two echocardiography assessments at least 6 months apart, and all echocardiographic examinations were performed when patients were in stable condition following treatment for HF. If a patient underwent more than two echocardiography tests, the baseline and final follow-up assessments were used to calculate changes in echocardiographic indices.

### Clinical Definitions

Diagnosis and classification of HF were made according to the 2022 AHA HF guidelines [2]. LVEF is critical for the diagnosis and classification of HF [HFpEF, LVEF ≥ 50%, HFmrEF, LVEF 40%–49%, and HFrEF, LVEF ≤ 40%]. An additional criterion for HFmrEF and HFpEF was objective evidence of spontaneous or provokable raised LV filling pressures, including increased natriuretic peptide levels and invasive/noninvasive hemodynamic measurement suggesting elevated LV filling pressures.

The diagnosis of AF required a single-lead ECG recording of ≥ 30 s or a 12-lead ECG showing heart rhythm with no discernible repeating P waves and irregular RR intervals (when atrioventricular conduction was not impaired). AF was classified as follows: Paroxysmal AF: AF that is self-terminating within 7 days or AF episodes terminated by intervention within 7 days. Persistent AF: continuous AF of > 7 days’ duration or AF terminated with cardioversion (drugs or direct current cardioversion) after 7 days or more [4].

Success in rhythm control was defined as freedom from any episode of atrial arrhythmia (AF, atrial flutter, or atrial tachycardia) within 12 months postoperatively. All patients undergoing CA were evaluated after a 3-month blanking period.

FMR and FTR were defined as the absence of any structural leaflet abnormality [13]. For the present analysis, patients graded none, and mild FMR/FTR was defined as ≤ mild FMR/FTR, while those with mild-moderate, moderate, moderate-severe, or severe FMR/FTR were defined as > mild FMR/FTR.

### Grouping, Study Endpoints, and Follow Up

Patients were stratified into the rhythm and rate control groups according to the type of treatment strategies for AF. In the HF subgroup analysis, the cohort was further divided into the HFrEF, HFmrEF, and HFpEF subgroups based on the LVEF measured at baseline. Additionally, clinical outcomes across the spectrum of HF subtypes were evaluated. Alterations in the structural parameters of the four cardiac chambers and LVEF from baseline to follow-up were assessed. The change in FMR/FTR severity was recorded. Effects on all-cause mortality and HF-related admission were analyzed.

12-lead ECG or Holter examinations were performed at 1, 3, 6, and 12 months or when symptoms relapsed in order to monitor the recurrence of atrial arrhythmias following rhythm control treatment.

Patients were required to make regular outpatient visits at 1, 3, 6, and 12 months and at 1-year intervals thereafter. If they did not attend scheduled clinic appointments, they were called for further interviews. The follow-up cut-off date was November 30, 2021 or the occurrence of death, whichever occurred first.

### Statistical Analysis

Statistical analysis was performed using Statistical Package for Social Sciences, version 24 (SPSS Inc., Chicago, IL, USA). Quantitative variables with non-normal distribution were described as median (interquartile range), and the Mann–Whitney U test was applied to assess the differences. Quantitative variables with normal distribution were expressed by means ± standard deviations and compared using the independent-sample t-test. Qualitative variables were presented as percentages (%), and differences between groups were analyzed by the chi-square test. Kaplan–Meier analysis was used to evaluate the incidence of pre-specified outcomes, with differences compared by means of the log-rank test. Univariate and multivariate Cox regression analyses were used for assessing the risk of all-cause mortality and HF-related admission across HF subgroups, and hazard ratios (HR), 95% confidence intervals (CI), and p values were calculated. Variables with known clinical significance and a p-value < 0.05 in univariable analyses were considered for inclusion in the multivariable Cox regression analysis. A two-sided p-value < 0.05 was considered statistically significant.

## Results

**Fig. 1:**
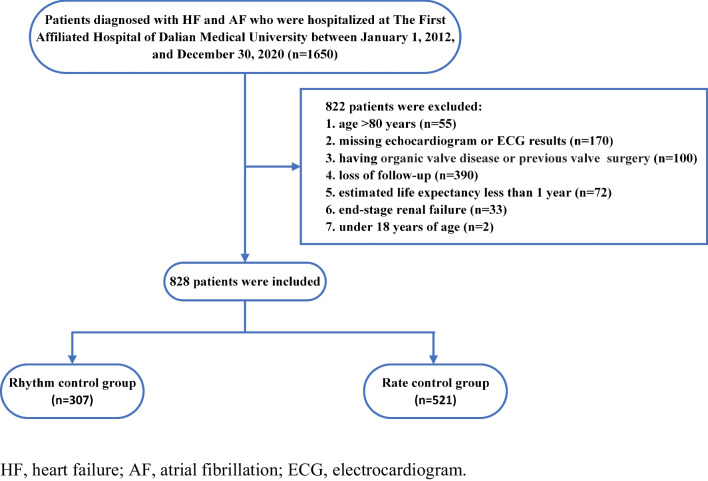
Flowchart of the study protocol

The flowchart for the identification, inclusion, and exclusion of study participants is illustrated in Fig. 1. Consequently, a total of 828 patients were included in the study; 307 patients underwent a strategy of rhythm control, and 521 patients underwent a strategy of rate control.

### Baseline Characteristics

The patients had a mean age of 67.00 ± 11.02 years old, and 54.8% were male; 475 had HFpEF [rhythm/rate control n = 165/310; median LVEF (%) 58.00 (55.00, 59.00)/57.00 (55.00, 58.00)], 93 had HFmrEF [rhythm/rate control n = 41/52; median LVEF (%) 45.00 (43.00, 47.00)/45.00 (44.00, 46.00)], and 260 had HFrEF [rhythm/rate control n = 101/159; median LVEF (%) 32.00 (30.00, 36.00)/33.00 (27.00, 37.00)] based on the baseline measurement of LVEF. In terms of their demographics, on the one hand, patients in the rhythm control group were younger and were more likely to have a higher heart rate and greater prevalence of New York Heart Association (NYHA) class II but less likely to have higher systolic blood pressure and CHA2DS2-VASc score. On the other hand, a history of ischemic heart disease, hypertension, diabetes, and persistent AF was less prevalent in the rhythm control group. Regarding medication, patients in the rhythm control group were less likely to be prescribed medications, including beta-blockers and antiplatelet therapies, whereas anticoagulant therapy was more frequent. In terms of laboratory tests, the rhythm control group had higher levels of hemoglobin but lower B-type natriuretic peptide levels. Regarding baseline echocardiographic variables, patients in the rhythm control group had lower values for LASID, LATD, LAV, LAVI, RASID, RATD, RVEDD, and were less likely to have > mild FMR/FTR (Table 1).

**Table 1:** Baseline characteristics of study participants

	Rhythm control group (n = 307)	Rate control group (n = 521)	P-value
Age (years)	63.66 (10.89)	68.96 (10.62)	< 0.001
Male (%)	179 (58.3)	275 (52.8)	0.123
NYHA class II (%)	100 (32.6)	115 (22.1)	0.001
NYHA class III (%)	166 (54.1)	307 (58.9)	0.173
NYHA class IV (%)	41 (13.4)	99 (19.0)	0.036
Left ventricular ejection fraction at baseline categories			0.181
HFrEF (%)	101 (32.9)	159 (30.5)	
HFmrEF (%)	41 (13.4)	52 (10.0)	
HFpEF (%)	165 (53.7)	310 (59.5)	
Median left ventricular ejection fraction at baseline (%)			
HFrEF	32.00 (30.00, 36.00)	33.00 (27.00, 37.00)	0.682
HFmrEF	45.00 (43.00, 47.00)	45.00 (44.00, 46.00)	0.509
HFpEF	58.00 (55.00, 59.00)	57.00 (55.00, 58.00)	0.093
Heart rate (b.p.m.)	90.00 (74.00, 116.00)	87.00 (71.00, 107.00)	0.027
Systolic blood pressure (mmHg)	126.98 (17.94)	136.77 (22.87)	< 0.001
Diastolic blood pressure (mmHg)	81.02 (14.42)	81.90 (14.72)	0.400
Ischemic heart disease (%)	51 (16.6)	138 (26.5)	0.001
Dilated cardiomyopathy (%)	26 (8.5)	44 (8.4)	0.991
Hypertrophic cardiomyopathy (%)	23 (7.5)	32 (6.1)	0.451
Hypertension (%)	166 (54.1)	329 (63.1)	0.010
Diabetes (%)	66 (21.5)	162 (31.1)	0.003
Previous stroke or TIA (%)	33 (10.7)	76 (14.6)	0.115
Paroxysmal atrial fibrillation (%)	94 (30.6)	112 (21.5)	0.003
Persistent atrial fibrillation (%)	213 (69.4)	409 (78.5)	0.003
CHA2DS2-Vasc score	3.23 (1.58)	3.88 (1.65)	< 0.001
Beta-blocker (%)	162 (52.8)	468 (89.8)	< 0.001
ACEI or ARB or ARNI (%)	139 (45.3)	260 (49.9)	0.198
Spirolactone (%)	180 (58.6)	319 (61.2)	0.461
Novel oral anticoagulants (%)	113 (36.8)	127 (24.4)	< 0.001
Warfarin (%)	162 (52.8)	190 (36.5)	< 0.001
Antiplatelet (%)	24 (7.8)	179 (34.4)	< 0.001
Hemoglobin (g/L)	140.00 (128.00, 153.00)	136.00 (122.00, 148.00)	< 0.001
BNP level (pg/mL)	345.00 (142.87, 600.91)	395.33 (190.45, 834.06)	0.004
Hs-TnI (ug/L)	0.02 (0.01, 0.05)	0.02 (0.01, 0.07)	0.073
Creatinine (mmol/L)	75.50 (63.00, 91.00)	76.00 (63.00, 95.40)	0.565
Uric acid (mmol/L)	396.00 (332.35, 481.00)	396.00 (324.00, 477.00)	0.704
Potassium (mmol/L)	4.06 (0.40)	4.02 (0.48)	0.215
LASID (mm)	62.00 (57.25, 67.00)	64.00 (59.00, 71.00)	< 0.001
LATD (mm)	47.00 (43.00, 51.00)	49.00 (45.00, 53.00)	< 0.001
LAV (ml)	65.74 (53.83, 83.61)	74.52 (58.34, 96.25)	< 0.001
LAVI (ml/m^2^)	37.88 (30.39, 46.47)	42.06 (32.80, 53.89)	0.001
RASID (mm)	55.00 (50.00, 59.00)	57.00 (51.50, 63.00)	< 0.001
RATD (mm)	42.00 (38.00, 45.00)	43.00 (38.00, 48.00)	< 0.001
LVEDD (mm)	50.00 (46.00, 57.00)	50.00 (46.00, 57.00)	0.964
RVEDD (mm)	18.00 (17.00, 20.00)	19.00 (17.00, 21.00)	< 0.001
≤ Mild FMR (%)	202 (65.8)	306 (58.7)	0.044
> Mild FMR (%)	105 (34.2)	215 (41.3)	0.044
≤ Mild FTR (%)	214 (69.7)	300 (57.6)	0.001
> Mild FTR (%)	93 (30.3)	221 (42.4)	0.001

Data are expressed as mean (standard deviation) or as median (Q1, Q3) or as number (percentage)

*NYHA* New York Heart Association, *TIA* transient ischemic attack, *ACEI* angiotensin-converting enzyme inhibitor, *ARB* angiotensin II receptor blocker, *ARNI* angiotensin receptor–neprilysin inhibitor, *BNP* B-type natriuretic peptide, *hs-TNI* high-sensitivity troponin I, *LASID* superior and inferior diameter of left atrium, *LATD* transverse diameter of left atrium, *LAV* left atrial volume, *LAVI* left atrial volume index, *RASID* superior and inferior diameter of right atrium, *RATD* transverse diameter of right atrium, *LVEDD* left ventricular end-diastolic dimension, *RVEDD* right ventricular end-diastolic dimension, *LVEF* left ventricular ejection fraction, *FMR* functional mitral regurgitation, *FTR* functional tricuspid regurgitation

In the rhythm control group, 192 (62.5%) and 115 (37.4%) underwent CA and ECV, and 133 (69.2%) and 56 (48.6%) patients maintained sinus rhythm (SR) within 12 months after rhythm control treatment, respectively.

### Subgroup Analysis

#### Changes in Cardiac Remodeling Parameters and LVEF between Two Groups

Compared to the rate control group, there was a greater reduction in biatrial size and improvements in LVEF in the rhythm control group. More specifically, regarding LA size, LATD and LAV were decreased in all subgroups of HF, including HFrEF [LATD (mm) –1.00 (–7.00, 2.00) vs. 0.00 (–4.00, 3.25), *p* = 0.036 and LAV (ml) –6.20 (–20.25, 7.64) vs. –2.48 (–14.22, 13.62), *p* = 0.046], HFmrEF [LATD (mm) –2.00 (–4.75, 2.00) vs. 1.00 (–3.00, 5.00), *p* = 0.025 and LAV (ml) –6.31 (–19.58, 7.31) vs. 2.60 (–4.77, 21.85), *p* = 0.008], and HFpEF [LATD (mm) 0.00 (–4.00, 3.00) vs. 2.00 (–2.00, 5.00), *p* < 0.001 and LAV (ml) –2.09 (–12.78, 7.52) vs. 7.39 (–3.97, 21.89), *p* < 0.001]; LASID was decreased in HFrEF [–3.00 (–7.50, 2.00) mm vs. 0.00 (–5.00, 3.00) mm, *p* = 0.010] and HFpEF [–1.00 (–6.00, 3.00) mm vs. 2.00 (–2.00, 7.00) mm, *p* < 0.001], but not in HFmrEF (*p* = 0.105). In addition, LAVI was similarly decreased in HFmrEF [–5.65 (–10.04, 2.04) ml/m^2^ vs. 1.27 (–2.55, 12.70) ml/m^2^, *p* = 0.006] and HFpEF [–0.77 (–8.80, 4.66) ml/m^2^ vs. 4.83 (–2.32, 12.44) ml/m^2^, *p* < 0.001], but not in HFrEF (*p* = 0.363). With regard to RA size, RASID was significantly reduced in HFrEF [–3.50 (–7.25, 1.00) mm vs. 0.00 (–6.00, 5.00) mm, *p* = 0.002], HFmrEF [–2.00 (–6.00, 4.00) mm vs. 2.50 (–2.00, 6.75) mm, *p* = 0.039], and HFpEF [0.00 (–4.50, 3.00) mm vs. 2.00 (–2.00, 6.00) mm, *p* < 0.001]. Likewise, RATD was significantly reduced in HFrEF [–2.00 (–7.00, 2.00) mm vs. –1.00 (–5.00, 4.00) mm, *p* = 0.018] and HFpEF [0.00 (–3.00, 4.00) mm vs. 2.00 (–2.00, 7.00) mm, *p* = 0.006], but with no significant difference in HFmrEF (*p* = 0.233). Regarding LVEF, the greatest difference was observed in HFrEF [7.00 (0.00, 17.50) % vs. 5.00 (–1.00, 14.00) %, *p* = 0.046]. However, there was no significant difference in HFmrEF (*p* = 0.742) and HFpEF (*p* = 0.133). Reductions in LVEDD and RVEDD were comparable across the HFrEF, HFmrEF, and HFpEF subgroups (Table 2).

**Table 2:** Changes in echocardiography parameters stratified by rhythm control and rate control among the HF subgroups

	Absolute change in cardiac structure parameters and LVEF		Comparison between treatment arms
	Rhythm control group	Rate control group	P-value
Patients with HFrEF			
LASID (mm)	–3.00 (–7.50, 2.00)	0.00 (–5.00, 3.00)	0.010
LATD (mm)	–1.00 (–7.00, 2.00)	0.00 (–4.00, 3.25)	0.036
LAV (ml)	–6.20 (–20.25, 7.64)	–2.48 (–14.22, 13.62)	0.046
LAVI (ml/m^2^)	–3.65 (–12.48, 4.58)	–2.10 (–9.96, 7.75)	0.363
RASID (mm)	–3.50 (–7.25, 1.00)	0.00 (–6.00, 5.00)	0.002
RATD (mm)	–2.00 (–7.00, 2.00)	–1.00 (–5.00, 4.00)	0.018
LVEDD (mm)	0.00 (–5.50, 3.00)	0.00 (–3.00, 3.50)	0.175
RVEDD (mm)	1.00 (–1.00, 2.00)	0.00 (–2.00, 1.00)	0.107
LVEF (%)	7.00 (0.00, 17.50)	5.00 (–1.00, 14.00)	0.046
Patients with HFmrEF			
LASID (mm)	–2.50 (–6.75, 3.00)	1.00 (–3.00, 5.00)	0.105
LATD (mm)	–2.00 (–4.75, 2.00)	1.00 (–3.00, 5.00)	0.025
LAV (ml)	–6.31 (–19.58, 7.31)	2.60 ( –4.77, 21.85)	0.008
LAVI (ml/m^2^)	–5.65 (–10.04, 2.04)	1.27 (–2.55, 12.70)	0.006
RASID (mm)	–2.00 (–6.00, 4.00)	2.50 (–2.00, 6.75)	0.039
RATD (mm)	0.00 (–4.00, 4.00)	1.00 (–3.00, 7.00)	0.233
LVEDD (mm)	0.00 (–3.00, 3.00)	0.00 (–4.00, 4.00)	0.648
RVEDD (mm)	0.00 ( –1.00, 1.00)	1.00 (–1.00, 2.00)	0.143
LVEF (%)	5.00 (–0.50, 10.00)	5.00 (–3.50, 10.00)	0.742
Patients with HFpEF			
LASID (mm)	–1.00 (–6.00, 3.00)	2.00 (–2.00, 7.00)	< 0.001
LATD (mm)	0.00 (–4.00, 3.00)	2.00 (–2.00, 5.00)	< 0.001
LAV (ml)	–2.09 (–12.78, 7.52)	7.39 (–3.97, 21.89)	< 0.001
LAVI (ml/m^2^)	–0.77 (–8.80, 4.66)	4.83 (–2.32, 12.44)	< 0.001
RASID (mm)	0.00 (–4.50, 3.00)	2.00 (–2.00, 6.00)	< 0.001
RATD (mm)	0.00 (–3.00, 4.00)	2.00 (–2.00, 7.00)	0.006
LVEDD (mm)	0.00 (–2.00, 3.00)	0.00 (–3.00, 3.00)	0.966
RVEDD (mm)	0.00 (–1.00, 2.00)	0.00 (–2.00, 2.00)	0.184
LVEF (%)	0.00 (–3.00, 1.00)	–1.00 (–4.00, 1.00)	0.133

Data are expressed as median (Q1, Q3)

*LVEF* left ventricular ejection fraction, *HFrEF* heart failure with reduced ejection fraction, *HFmrEF* heart failure with mildly reduced ejection fraction, *HFpEF* heart failure with preserved ejection fraction, *LASID* superior and inferior diameter of left atrium, *LATD* transverse diameter of left atrium, *LAV* left atrial volume, *LAVI* left atrial volume index, *RASID* superior and inferior diameter of right atrium, *RATD* transverse diameter of right atrium, *LVEDD* left ventricular end-diastolic dimension, *RVEDD* right ventricular end-diastolic dimension

### Change in FMR and FTR among the HF Subgroups

**Fig. 2:**
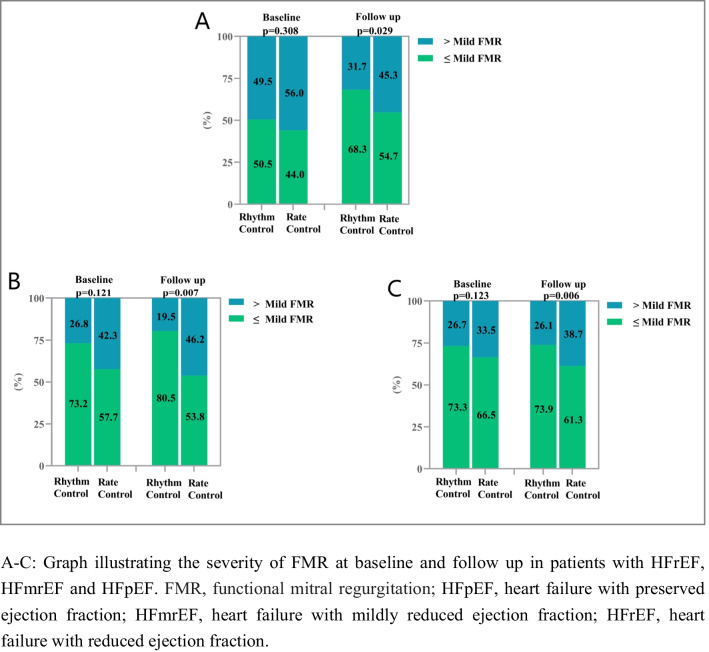
The severity of FMR at baseline and follow up among the HF subgroups

Both groups had similar percentages of patients with ≤ mild and > mild FMR at baseline across the HF categories (*p* > 0.05). At follow-up, patients in the rhythm control group had significantly less > mild FMR than patients in the rate control group across all subgroups of HF, including HFrEF (31.7% vs. 45.3%, *p* = 0.029), HFmrEF (19.5% vs. 46.2%, *p* = 0.007) and HFpEF (26.1% vs. 38.7%, *p* = 0.006) (Fig. 2).

**Fig. 3:**
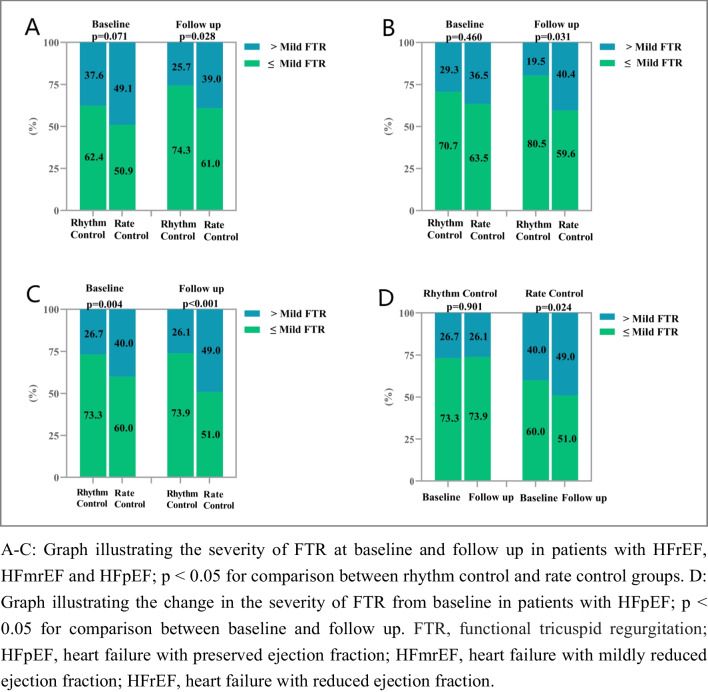
The severity of FTR at baseline and follow up among the HF subgroups

In HFrEF and HFmrEF, there was a lower proportion of > mild FTR at follow-up in the rhythm control group compared to the rate control group [HFrEF (rhythm control vs. rate control 25.7% vs. 39.0%, *p* = 0.028); HFmrEF (rhythm control vs. rate control 19.5% vs. 40.4%, *p* = 0.031)], despite the proportion of > mild FTR being similar at baseline (*p* > 0.05). Meanwhile, in HFpEF, there was a statistically significant difference in the degree of FTR between the two groups at baseline (*p* = 0.004). At follow-up, there was no significant difference in the degree of FTR compared to baseline in the rhythm control group (*p* = 0.901), but a significant increase in the degree of FTR was observed in the rate control group [percentage of > mild FTR: baseline vs. follow up: 40.0% vs. 49.0%, *p* = 0.024] (Fig. 3).

#### Adverse Outcomes Stratified by Rhythm and Rate Control

**Fig. 4:**
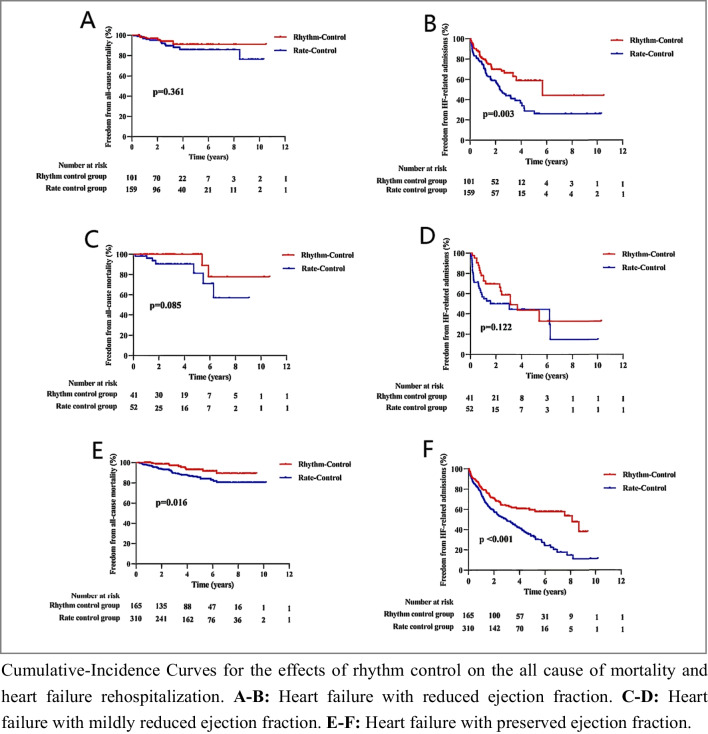
Adverse outcomes stratified by rhythm and rate control among the HF subgroups

The 828 patients were followed up for a maximum of 10.7 years and a median of 3.8 years. During this period, a total of 81 patients died; 21 in HFrEF [rhythm/rate control 6 (5.9%)/15 (9.4%)], 9 in HFmrEF [rhythm/rate control 2 (4.9%)/7 (13.5%)], and 51 in HFpEF [rhythm/rate control 10 (6.1%)/41 (13.2%)]; 404 patients required hospitalization attributable to HF; 112 in HFrEF [rhythm/rate control 33 (32.7%)/79 (49.7%)], 47 in HFmrEF [rhythm/rate control 19 (46.3%)/28 (53.8%)], and 245 in HFpEF [rhythm/rate control 64 (38.8%)/181 (58.4%)]. Kaplan–Meier curves and log-rank tests for mortality and HF rehospitalization across HF subgroups are delineated in Fig. 4. The unadjusted and adjusted Cox models among the HFrEF, HFmrEF, and HFpEF subgroups are presented in Table 3. In HFrEF, rhythm control was associated with lower risks of hospitalization for worsening HF (HR 0.500 [95%CI 0.330–0.757], *p* = 0.001), but the risk for all-cause mortality was similar to the rate control group; these correlations were similar after adjusting for multiple confounders. Contrastingly, in HFmrEF, there was no significant difference in the rhythm control group in terms of all-cause mortality and HF-related admission, and the difference was not statistically significant after adjusting for potential confounders. Finally, in HFpEF, the rhythm control group experienced lower risks of all-cause mortality (HR 0.436 [95% CI 0.218–0.871], *p* = 0.019) and HF-related admission (HR 0.541 [95% CI 0.407–0.720], *p* < 0.001), and remained significant after adjusting for multiple potential confounders.

**Table 3:** Risks of adverse outcomes stratified by rhythm control and rate control among the HF subgroups

	Model 1		Model 2	
Adverse outcome	HR (95%CI)	P value	HR (95%CI)	P-value
Patients with HFrEF				
All-cause mortality	0.644 (0.248–1.670)	0.365	0.994 (0.339–2.917)	0.992
HF rehospitalization	0.500 (0.330–0.757)	0.001	0.638 (0.410–0.993)	0.047
Patients with HFmrEF				
All-cause mortality	0.273 (0.056–1.323)	0.107	0.138 (0.014–1.384)	0.092
HF rehospitalization	0.630 (0.348–1.138)	0.126	0.842 (0.357–1.989)	0.695
Patients with HFpEF				
All-cause mortality	0.436 (0.218–0.871)	0.019	0.389 (0.162–0.938)	0.035
HF rehospitalization	0.541 (0.407–0.720)	< 0.001	0.554 (0.392–0.782)	0.001

Model 1: unadjusted. Model 2: adjusted for age, ischemic heart disease, history of hypertension, history of diabetes, NYHA class IV, use of ACEI or ARB or ARNI at baseline, use of beta-blocker at baseline, use of spironolactone at baseline, use of ACEI or ARB or ARNI during follow up, use of beta-blocker during follow up, use of spironolactone during follow up, use of antiplatelet, and serum potassium levels. *HR*, hazard ratio; *CI*, confidence interval; *HF*, heart failure; *HFrEF*, heart failure with reduced ejection fraction; *HFmrEF*, heart failure with mildly reduced ejection fraction; *HFpEF*, heart failure with preserved ejection fraction; *NYHA*, New York Heart Association; *ACEI*, angiotensin-converting enzyme inhibitor; *ARB*, angiotensin II receptor blocker; *ARNI*, angiotensin receptor–neprilysin inhibitor

## Discussion

Herein, rhythm control therapy (1) improved biatrial remodeling and LVEF; (2) decreased the severity of FMR and FTR; (3) reduced the risk of death in patients with HFpEF and the risk of HF rehospitalization in patients with HFrEF and HFpEF.

### Effects of Rhythm Control on Cardiac Remodeling

Transthoracic echocardiography was the primary noninvasive cardiac imaging method for assessing cardiac structure and function in patients with AF and HF. To date, the majority of studies on alterations in the cardiac anatomy and functions following rhythm control in patients with HF and AF have focused on LVEF, with only limited data on the structural alterations in all cardiac chambers, including LA, RA, LV, and RV [9–11, 14–17].

AF and HF, especially HFpEF, were associated with adverse atrial remodelling reflective of an underlying atrial disease process, which main pathophysiological mechanism was ion-channel changes and atrial fibrosis. AF-induced atrial myopathy has alterations that rely on AF duration. For example, very short-term AF does not produce ultrastructural changes, whereas AF lasting several weeks results in EHRAS I changes. Long-term persistent AF produces EHRAS III alterations [18–21]. Clinically, it has been reported that restoration and maintenance of SR (even by cardioversion) could result in a decrease in the atrial size [22, 23]. Moreover, LA enlargement was the strongest independent predictor of new-onset AF, AF recurrence, stroke, and worsening HF [24–27]. Meanwhile, higher RA volume was associated with an elevated risk of incident AF [28, 29]. In this study, a reduction in biatrial size and an improvement in LVEF were detected following rhythm control, which highlights the beneficial effects of rhythm control in patients with HF and AF. Therefore, the option of active rhythm control for AF could be discussed with patients in HF to reduce the cardiac size and avoid the worsening of both atrial and ventricular dysfunction. It is noteworthy to point out that data regarding the effect of rhythm control on RV and LV size in AF are scarce. In this study, there was no significant difference in changes in ventricular diameter between the two groups, which could be explained by previous research showing that ventricular reversal occurs later than the reversal of atrial dimensions following CA in persistent AF. These results were also consistent with the findings of the CAMERA-MRI study, which reported that CA caused greater improvement in LVEF, but no significant reduction in LVEDD in patients with systolic dysfunction [11].

### Effects of Rhythm Control on Functional Regurgitation

AF leads to biatrial dilatation, potentially with annular dilatation of the atrioventricular valves, thereby subsequently promoting the development of FMR and FTR [12]. Therefore, rhythm control for AF could improve adverse remodeling of the atrium and functional regurgitation. This hypothesis was validated by Soulat-Dufour et al., who identified a decrease in FMR and FTR after the restoration of SR in AF [22]. In patients with HF and comorbid AF, an improvement was noted in FMR and FTR among all classes of HF, which may be related to the reverse remodeling in the cardiac chambers following rhythm control.

### Effects of Rhythm Control on Prognosis

The famous CASTLE-AF trial demonstrated that CA treatment resulted in a marked reduction in the composite endpoint of all-cause mortality or HF-related admission compared with medical therapy in patients with HFrEF [9], but the trial results in outcomes were heterogeneous, and the available evidence showed limited benefits from CA among patients with more advanced HF and more severe LV dysfunction [14, 30]. In the present study, 85% of patients in the HFrEF subgroup suffered from NYHA class III or IV HF. Our results showed that rhythm control led to a lower incidence of hospitalization for worsening HF but no significant reduction in all-cause mortality. This neutral result in survival was also similar to previously published data [14, 30]. At present, most studies have focused on AF and HFrEF [9], and few studies have compared the effects of rhythm control and rate control therapies in HFmrEF or HFpEF. In the EAST-AFNET4 trial, 798 patients diagnosed with HF and AF were enrolled. In this cohort, the percentages of HFpEF, HFmrEF, and HFrEF were 442 (55.4%), 211 (26.4%) and 132 (16.5%), respectively. The study showed that early rhythm control reduced a composite primary end point of acute coronary syndrome or HF admission compared with conventional treatment in all the HF patients. Although the HF subgroups tended to benefit from early rhythm control, there was no statistically significant difference. Unlike the EAST-AFNET4 trial, in this HFpEF subgroup, favorable effects of rhythm control were also observed on survival and HF-related admission [16], which was consistent with the former studies that have depicted favorable outcomes following rhythm control for AF in patients with HFpEF [10, 31]. Therefore, the results reported in our study provide further evidence of the clinical benefits of rhythm control in HFpEF patients and that the choice of AF therapy should be incorporated into risk stratification strategies for HF.

### Limitations

The limitations of this study should also be taken into account when interpreting the results. First, this was a single-center, retrospective, and observational study with selection and recall bias. Another limitation was the lack of a standardized strategy to monitor arrhythmia recurrence (e.g., implantable loop recorder).

## Conclusions

A rhythm control strategy in patients with HF and comorbid AF resulted in enhanced cardiac remodeling, functional regurgitation, and prognosis compared with rate control. Thus, these data signal a beneficial effect of rhythm control in patients with HF and comorbid AF.

## Data Availability

The original contributions presented in the study are included in the article/Supporting Information Material, further inquiries can be directed to the corresponding authors.
